# Why Me? To Be an Ultra-Responder to Antiplatelet Therapy: A Case Report

**DOI:** 10.3389/fneur.2021.663308

**Published:** 2021-08-10

**Authors:** Francesca Rosafio, Guido Bigliardi, Nicoletta Lelli, Laura Vandelli, Federica Naldi, Ludovico Ciolli, Stefano Meletti, Andrea Zini

**Affiliations:** ^1^Stroke Unit, Neurology Unit, Department of Neuroscience, Ospedale Civile Baggiovara, Azienda Ospedaliera Universitaria di Modena, Modena, Italy; ^2^Laboratory of Clinical Pathology and Toxicology, Department of Laboratory Medicine, Ospedale Civile Baggiovara, Azienda Ospedaliera Universitaria di Modena, Modena, Italy; ^3^Istituto di Ricovero e Cura a Carattere Scientifico (IRCCS) Istituto delle Scienze Neurologiche di Bologna, Department of Neurology and Stroke Center, Maggiore Hospital, Bologna, Italy; ^4^Department of Biomedical, Metabolic and Neural Sciences, University of Modena and Reggio Emilia, Modena, Italy

**Keywords:** case report, aggregometry, antiplatelet therapy, primary prevention, intracerebral hemorrhage

## Abstract

**Background:** Platelet function testing is a valid tool to investigate the clinical response to antiplatelet therapy in different clinical settings; in particular, it might supply helpful information in patients with cerebrovascular disease. Oral antiplatelet treatment, such as Aspirin (ASA) and Clopidogrel, is the gold standard in secondary stroke prevention of non-cardiogenic ischemic stroke; conversely, its application as a primary prevention therapy is not routinely recommended in patients with vascular risk factors. Multiple electrode platelet aggregometry (MEA) impedance aggregometer is a validated device to test platelet inhibition induced by ASA or Clopidogrel.

**Case Report:** We report the case of a 78-year-old patient without relevant clinical history, taking ASA as primary prevention strategy, who was admitted for sudden onset of dysarthria and left facial hyposthenia during physical effort. Brain CT revealed two small subcortical bilateral spontaneous intracranial hemorrhages. Platelet aggregometry with MEA performed upon admission revealed a very strong platelet inhibition induced by ASA (result of the ASPI Test was 5 U, consistent with an ultra-responsiveness to ASA, and the cutoff value of correct responsiveness is <40 U). MRI at longitudinal follow-up revealed the presence of two small cavernous angioma underlying hemorrhagic spots.

**Conclusion:** The evaluation of platelet reactivity in stroke patients undergoing antiplatelet therapies, not commonly performed in clinical practice, could be useful to optimize prevention strategies; the verification of the biological effectiveness of ASA or Clopidogrel could be a valid tool in the definition of each patient's risk profile, particularly in patients with cerebrovascular disease known to be at increased risk for both hemorrhagic and thrombotic complications.

## Background

International leading guidelines strongly recommended antiplatelet therapy in secondary prevention of non-cardiogenic strokes, as it is associated with an estimated reduction of relative risk of stroke or death on average by about 22% (1, 2). Conversely, the use of pharmacological strategy for primary cardiovascular prophylaxis, including stroke prevention, is still a debated topic (3). It is mandatory to improve the control of modifiable risk factors, such as hypertension and diabetes, but antiplatelet agents have no clear indications (3). Recently, the ACC/AHA guideline suggests to address primary prevention with low-dose Aspirin daily treatment to selected patients between 40 and 79 years of age, who are at higher risk for ischemic vascular event, but not at increased bleeding risk (4).

Therefore, the use of Aspirin might be reasonable only for people whose 10-year vascular risk is notable (at least higher than 10%) for the benefits to outweigh the risks associated with treatment. In particular, the association of diabetes mellitus with other high-risk conditions has been considered for primary prevention strategies (1, 3, 4).

Platelet function testing is a valid tool to investigate the clinical response to antiplatelet therapy in different clinical settings; several clinical and biological mechanisms for antiplatelet “resistance” or, conversely, “ultra-responsiveness” have been supposed (incongruent dose, poor compliance, genetic polymorphisms, baseline hyperactivity, and/or accelerated platelet turnover) (5–7). Thus, the possibility of testing the biological effectiveness of antiplatelet medications in vascular patients could be potentially useful for promptly detecting any relevant clinical problems, including safety in ultra-responder patients (8). However, the implementation of platelet function testing in routine clinical practice is not widely supported, mainly due to a lack of consensus on the effective improvement of clinical outcome with tailored therapy; other studies conversely debated the usefulness of platelet function monitoring, particularly in terms of reliability of results between different tests available (9, 10).

**Figure 1:**
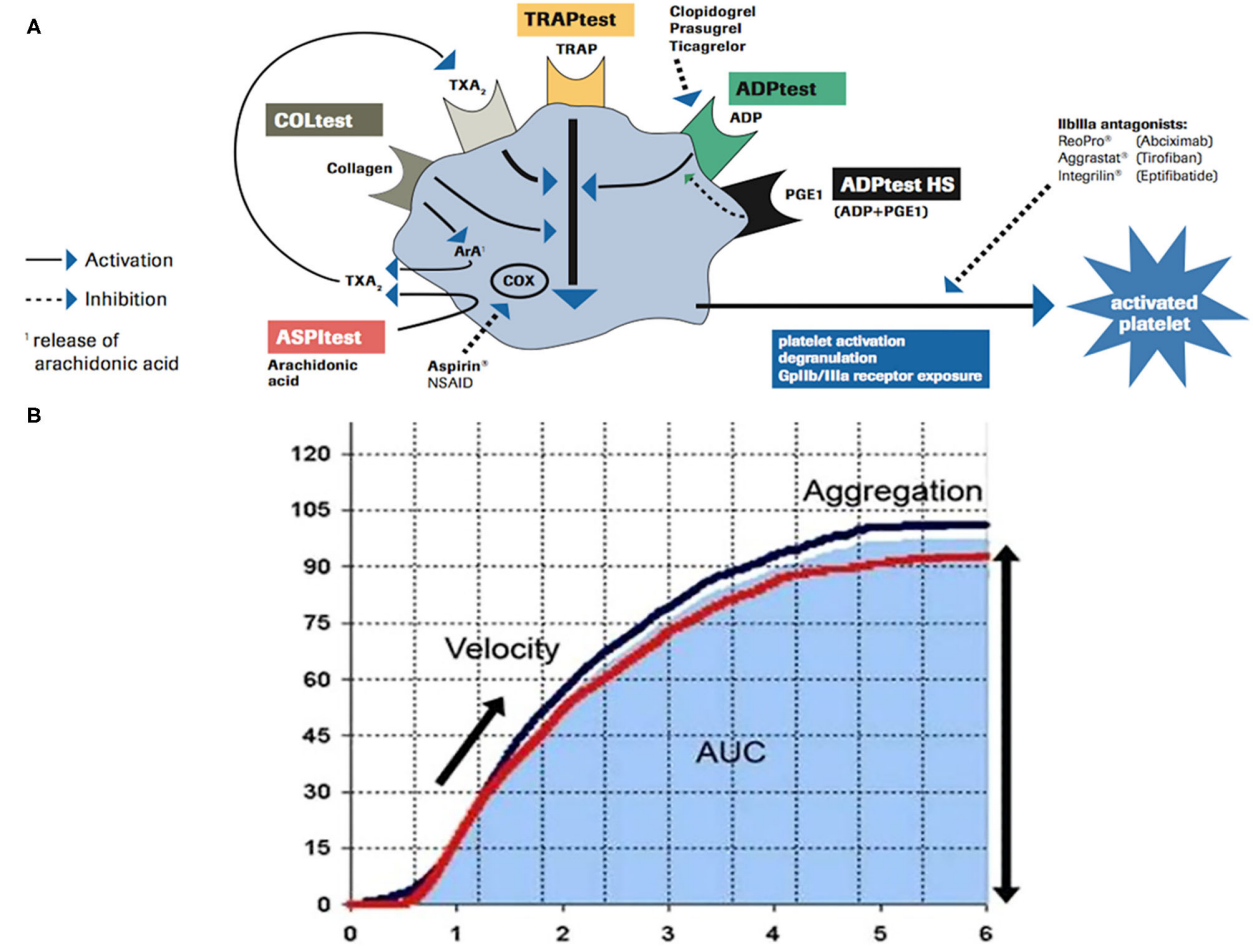
**(A)** Schematic overview of platelet activation/inhibition pathways and impedance aggregometry tests (ADP Test, ASPI Test, and TRAP Test). **(B)** Graphic presentation of platelet–platelet aggregation induced during each test; platelet responsiveness is quantified by the area under the curve (AUC*min). Modified from Roche Diagnostics International.

Within impedance aggregometers, the device “Multiple Electrode Platelet Aggregometry” (MEA, Multiplate Analyzer^®^, Roche Diagnostics International Ltd., CH-6343 Rotkreuz, Switzerland) (11, 12) showed correlation with the estimates of the antiplatelet effect of Clopidogrel and ASA obtained by other methods (13). Platelet aggregometry is a function test based on the stimulation of platelet–platelet aggregation with various agonists [adenosine diphosphate (ADP), arachidonic acid (ASPI), and thrombin receptor-activating peptide (TRAP)] and can be used to monitor the effects of antiplatelet agents, classified into three groups regarding their mechanism of action (thromboxane inhibitors—Aspirin, ASA, ADP receptor antagonists—Clopidogrel, and glycoprotein IIb/IIIa inhibitors). A comprehensive overview of platelet activation pathways is summarized in Figure 1A. According to the principles of impedance aggregometry, Multiplate Analyzer^®^ assessed residual platelet function in whole blood of patients undergoing antiplatelet therapy; every test is performed in a single-use test cell, which incorporates two independent impedance metal sensors. After the addition of specific agonists (ADP, ASPI, and TRAP), the platelet–platelet aggregation is induced and real-time recording starts. The ADP Test reagent contains ADP, which triggers platelet activation *via* different ADP receptors, the most important of which is blocked by Clopidogrel (14). The ASPI Test reagent contains arachidonic acid, whose activation pathway is blocked by ASA (15); TRAP aggregation test is used to obtain a platelet aggregation measure relatively independent of others, supporting the proper sample preparation. Once activation of platelet aggregation starts on metal sensors, the electrical resistance increases; the resistance change is transformed to arbitrary aggregation units (AUs) and plotted against time. The area under the aggregation curve (AUC) quantifies the aggregation response, expressed in units (U; 1 U corresponds to 10 AU^*^min) (Figure 1B). Cutoff value of the ASPI Test indicating correct responsiveness to ASA is <40 U (16), while values under 30 U indicate strong enzymatic inhibition and higher risk of bleeding (17).

## Case Presentation

A 78-year-old man without relevant clinical history was admitted in the Stroke Unit for sudden onset of slurred speech and left oral rhyme deviation during physical effort, without headache and/or limb weakness. Patient's past medical history reported bilateral neurosensory hypoacusis, previous cataract surgery, and carpal tunnel syndrome surgically treated. Pharmacological anamnesis revealed daily treatment with Aspirin 100 mg as a vascular primary prevention strategy, started 3 months before.

**Figure 2:**
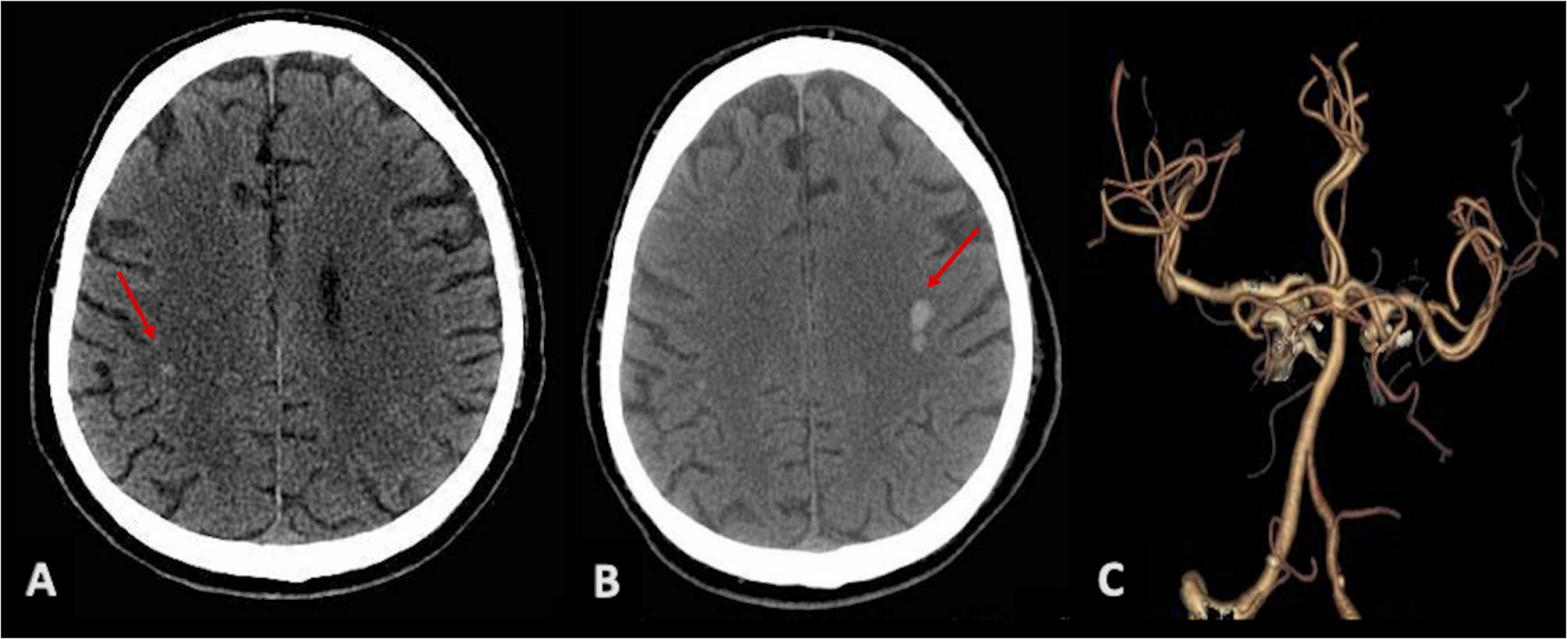
Brain CT scans showing bilateral intraparenchymal hyperdense lesions (red arrows): a small hemorrhagic spot in the post-rolandic area on the right **(A)** and a greater hematoma in the left pre-rolandic area **(B)**. **(C)** CTA with no evidence of vascular malformations.

Neurological examination showed paralysis of right VII cranial nerve, right deviation of protruded tongue, and mild dysarthria (NIH stroke scale 2/42). Brain CT revealed multiple chronic lacunar infarctions of basal ganglia bilaterally, and two acute small intraparenchymal hemorrhages, within post-rolandic subcortical region on the right side (Figure 2A) and pre-rolandic subcortical region on the left side (Figure 2B); CT angiography showed mild carotid and vertebral atherosclerosis, and no vascular malformation (Figure 2C).

**Figure 3:**
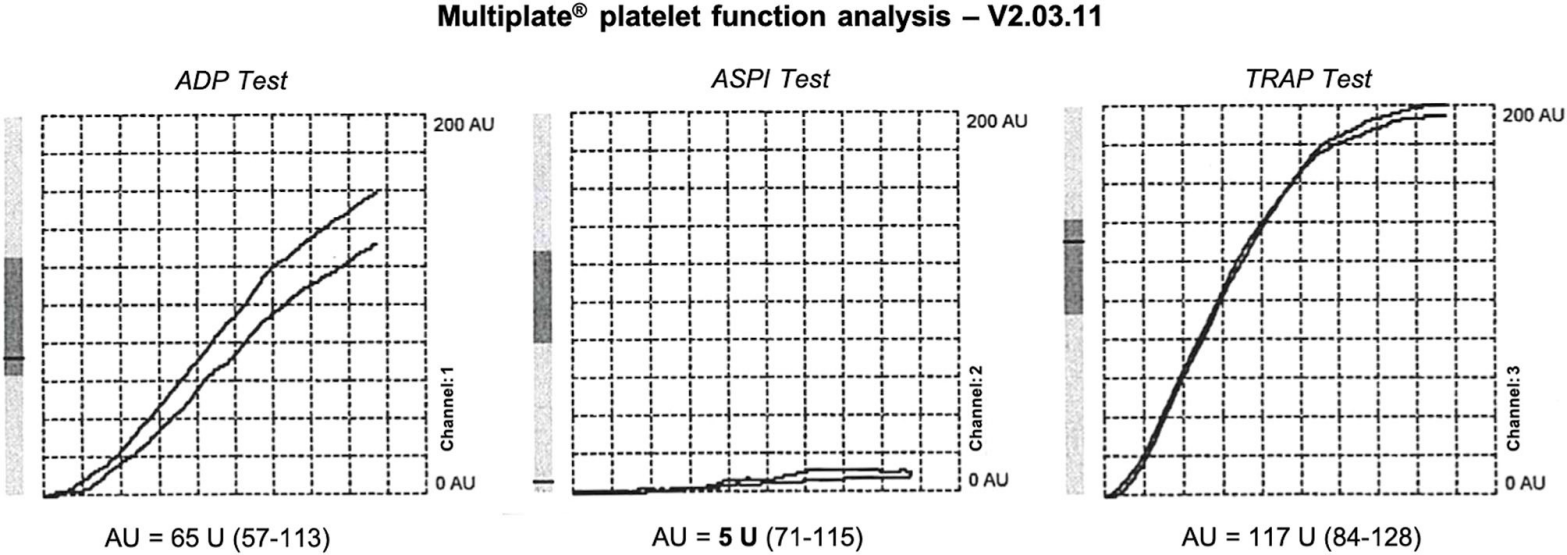
Platelet function testing performed upon admission during Aspirin therapy. Marked reduction of AUC on ASPI Test, 5 U, expressing a strong platelet inhibition induced by ASA (ultra-responder patient). Expected values in healthy individuals are in brackets.

Aspirin therapy was immediately discontinued. Intensive monitoring in the Stroke Unit and cardiac ultrasound revealed an unknown arterial hypertension, with a chronic hypertensive cardiopathy. Target therapy with ACE inhibitors (Enalapril 20 mg once daily) was started, with blood pressure normalization. Multiplate^®^ platelet function analysis performed upon admission revealed a very strong platelet inhibition induced by ASA; the area under the aggregation curve (AUC) on the ASPI Test was 5 U, consistent with an ultra-responsiveness to ASA, with normal platelet aggregation induced by other agonists on the ADP Test and TRAP Test (Figure 3).

**Figure 4:**
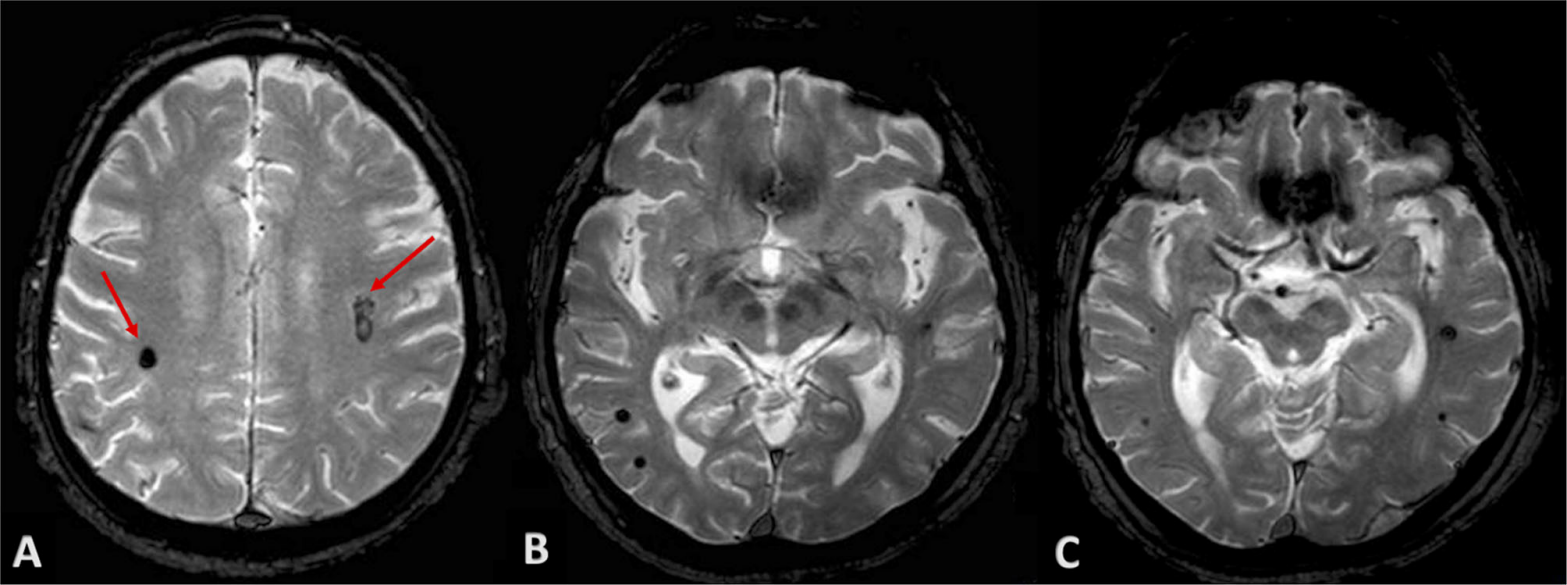
Patient's MRI at follow-up. Gradient-echo T2*-weighted sequences revealed roundish lesions underlying well-known intraparenchymal hemorrhages (**A**, red arrows), with classic magnetic resonance appearance of cavernous venous malformations. **(B,C)** show multiple and similar lesions throughout subcortical white matter and basal ganglia, bilaterally.

Neurological examination of patients at discharge was completely normalized. Due to the “atypical” locations of intraparenchymal hematomas, we performed a brain MRI at longitudinal follow-up in order to exclude non-hypertensive causes of bleeding. Gradient-echo T2^*^-weighted sequences revealed two small roundish lesions, in the anatomical site of bilateral subcortical hematomas, with minute central nucleus of methemoglobin and dark hemosiderin rim, and without surrounding edema, consistent with cavernous venous malformations (Figure 4A). Multiple similar but smaller cavernous angiomas were detected throughout subcortical white matter on both sides, particularly in temporal and occipital lobes, and in the area of basal ganglia (Figures 4B,C). On differential diagnosis, T2 and FLAIR sequences excluded findings suggestive of other conditions, as possible cerebral amyloid angiopathy; no evidence of significant subcortical leukoencephalopathy was detected besides lacunar microinfarcts in the region of basal ganglia bilaterally, and no signs consistent with superficial siderosis were detected.

## Discussion

We presented a case of a previous healthy patient, admitted for intracerebral atypical hemorrhages, taking no medications except ASA in primary prevention. Diagnostic workup revealed a condition of unrecognized arterial hypertension, and the presence of multiple intracerebral cavernous venous malformations, some of which with acute bleeding. Symptomatic hemorrhagic complication occurs as a clinical manifestation of cavernous angioma in 25% of cases (18), but the annual average rate of bleeding is reported to be lower in patients without history of prior hemorrhage (19). However, rupture rate rises in patients with associated condition at risk of bleeding, such as hypertension. Many studies suggest the likely safety of antiplatelet medications in patients with cerebral cavernous malformations (19), but outside of randomized controlled protocols (20).

The role of antiplatelet agents for the primary prevention of cardiovascular disease, including stroke, is still widely debated, due to the delicate balance between efficacy and safety in patients without established previous vascular events. Several randomized clinical trials showed that Aspirin is effective in the reduction of recurrence risk, with a tolerable increase of bleeding complications; thus, international practice guidelines strongly recommended ASA in secondary prevention of vascular diseases, as ischemic stroke or myocardial infarction (1). Regarding primary prevention, diverging results have contributed to unclear indications about antiplatelet therapy, which is not routinely recommended, primarily due to safety (4). Therefore, in clinical practice, ASA treatment should be tailored on each patient's risk profile (e.g., associations of diabetes mellitus and other high-risk conditions) and might be reasonable only in case of a notable 10-year risk of primary vascular events (3, 4).

The possibility to test the biological effectiveness of antiplatelet agents, with platelet function testing devices such as Multiplate Analyzer^®^, might supply helpful information to clinicians, primarily to assess the responsiveness to ASA or Clopidogrel in ischemic stroke patients. Nevertheless, it might be a valid tool in the stratification of patient's risk profile as well, while considering the safety of a primary prevention regimen, particularly in the presence of clinical conditions associated with an increased risk of hemorrhagic complications.

However, longitudinal studies are needed to assess whether aggregometry might supply individualized information and whether it can be considered a valid tool in the development of tailored therapies, as the main limitation of its implementation in everyday clinical practice.

## Conclusion

Our report illustrates the potential clinical benefit of platelet function testing in patients undergoing antiplatelet therapy, with particularly useful application in the definition of patient's risk profile in case of primary prevention treatment with Aspirin. However, RCTs and longitudinal studies are needed to assess whether routine platelet function monitoring might be considered a decision-making tool for clinicians, both in patients with vascular diseases subjected to secondary prevention therapy and during the evaluation of safety profile of antiplatelet treatment in selected patients deserving of pharmacological primary prevention therapy.

## Data Availability Statement

The raw data supporting the conclusions of this article will be made available by the authors, without undue reservation.

## Ethics Statement

Written informed consent was obtained from the individual for the publication of any potentially identifiable images or data included in this article.

## Author Contributions

FR, GB, and AZ: manuscript drafting/revising, data acquisition, and final revision. NL: data acquisition. LV: manuscript revising and data acquisition. FN: manuscript revising. LC: manuscript revising and data acquisition. SM: manuscript drafting/revising and final revision. All authors approved the final version.

## Conflict of Interest

AZ has received funding for speaker honoraria and consulting fees from Boehringer-Ingelheim and speaker honoraria from Cerenovus. The remaining authors declare that the research was conducted in the absence of any commercial or financial relationships that could be construed as a potential conflict of interest.

## Publisher's Note

All claims expressed in this article are solely those of the authors and do not necessarily represent those of their affiliated organizations, or those of the publisher, the editors and the reviewers. Any product that may be evaluated in this article, or claim that may be made by its manufacturer, is not guaranteed or endorsed by the publisher.
